# A scoping review on the use of consumer-grade EEG devices for research

**DOI:** 10.1371/journal.pone.0291186

**Published:** 2024-03-06

**Authors:** Joshua Sabio, Nikolas S. Williams, Genevieve M. McArthur, Nicholas A. Badcock

**Affiliations:** 1 School of Psychology, University of Queensland, St Lucia, Queensland, Australia; 2 School of Psychological Science, University of Western Australia, Perth, Western Australia, Australia; 3 School of Psychological Science, Macquarie University, Sydney, New South Wales, Australia; 4 Emotiv Inc., San Francisco, California, United States of America; Nanyang Technological University, SINGAPORE

## Abstract

**Background:**

Commercial electroencephalography (EEG) devices have become increasingly available over the last decade. These devices have been used in a wide variety of fields ranging from engineering to cognitive neuroscience.

**Purpose:**

The aim of this study was to chart peer-review articles that used consumer-grade EEG devices to collect neural data. We provide an overview of the research conducted with these relatively more affordable and user-friendly devices. We also inform future research by exploring the current and potential scope of consumer-grade EEG.

**Methods:**

We followed a five-stage methodological framework for a scoping review that included a systematic search using the Preferred Reporting Items for Systematic Reviews and Meta-Analyses Extension for Scoping Reviews (PRISMA-ScR) guidelines. We searched the following online databases: PsycINFO, MEDLINE, Embase, Web of Science, and IEEE Xplore. We charted study data according to application (BCI, experimental research, validation, signal processing, and clinical) and location of use as indexed by the first author’s country.

**Results:**

We identified 916 studies that used data recorded with consumer-grade EEG: 531 were reported in journal articles and 385 in conference papers. Emotiv devices were used most, followed by the NeuroSky MindWave, OpenBCI, interaXon Muse, and MyndPlay Mindband. The most common usage was for brain-computer interfaces, followed by experimental research, signal processing, validation, and clinical purposes.

**Conclusions:**

Consumer-grade EEG is a useful tool for neuroscientific research and will likely continue to be used well into the future. Our study provides a comprehensive review of their application, as well as future directions for researchers who plan to use these devices.

## Introduction

Electroencephalography (EEG) is the continuous measurement of electrical activity generated by neurons firing in the brain. This involves placing metal electrodes at multiple scalp sites to record fluctuations in voltage at millisecond-level temporal resolution. Such recordings can then be processed to produce spectral analyses of electrical activity or generate event-related potentials (ERPs) that represent the averaged response to a task or stimulus. Today, EEG is one of the most popular neuroscientific tools for academics and medical professionals due to its non-invasiveness and ease-of-use [1]. More recently, several companies have developed consumer-grade EEG devices. These devices are compact, wireless, and have a streamlined setup, making them particularly attractive to novice researchers or those looking to collect data outside the traditional laboratory setting [2]. More importantly, consumer-grade devices are cheaper than research-grade devices, allowing those with limited funding an affordable means to collect neurophysiological data.

Due to its accessibility, consumer-grade EEG has been used for a wide variety of purposes across different fields. Software engineers and computer scientists use consumer-grade EEG to collect high resolution time-series data. This data is then processed to create or optimise machine learning and signal processing algorithms [3–5]. In turn, these algorithms can be used in conjunction with the device to develop brain-computer interface (BCI) systems. Those in engineering and robotics can train machines to respond in real time to patterns in neural data [6]. Once synchronised, a human user can configure a BCI to control a multitude of electronic devices including wheelchairs [7], drones [8], smart homes [9–11], and web browsers [12]. Clinicians report using the technology to administer neurofeedback therapy [13], facilitate learning [14], assess patient sleep quality [15, 16], and determine affective states [17–20]. Scientists increasingly use consumer-grade devices to collect neural data to address a variety of theoretical and practical research questions [2, 21, 22].

The proliferation of research with consumer-grade EEG has inspired several non-systematic reviews (see Table 1). For instance, some reviews compared the performance of a single consumer-grade EEG device to non-EEG biosensors in the domains of seizure detection [23], BCI systems [24], and stress recognition [25]. Other reviews have compared *multiple* consumer-grade EEG devices within a single domain [2, 21, 26–28]. For instance, Dadebayev et al. [29] focused their review on emotion recognition; Asl et al. [30] focused on drowsiness detection and Khurana et al. [31] focused on neuromarketing. One of the most thorough reviews considered around 100 “handpicked” [22] studies that used four consumer-grade devices–the NeuroSky MindWave, Emotiv EPOC+, interaXon Muse, and OpenBCI neuroheadset–in the domains of cognition, BCI, education research, and game development.

**Table 1:** List of literature reviews that include consumer-grade EEG devices.

First Author	Year	Domain	Devices Reviewed
Rechy-Ramirez et al.	2018	BCI	Emotiv
Xu et al.	2018	Educational research	Emotiv; NeuroSky MindWave
Aldridge et al.	2019	P300 speller task	OpenBCI
Kurada et al.	2019	Seizure detection	Emotiv
Roy et al.	2019	Deep learning	Emotiv; interaXon Muse; Myndplay Mindband; NeuroSky MindWave; OpenBCI
LaRocco et al.	2020	Drowsiness detection	Emotiv; interaXon Muse; NeuroSky MindWave; OpenBCI
Vasiljevic et al.	2020	BCI gamification	Emotiv; Myndplay Mindband; NeuroSky MindWave; OpenBCI
Shum et al.	2021 [33]	Seizure detection	Emotiv; NeuroSky MindWave
Castro-Garcia et al.	2022	Stress recognition	OpenBCI
Khurana et al.	2021	Neuromarketing	Emotiv; NeuroSky MindWave; MyndPlay Mindband; OpenBCI
Dadebayev et al.	2022	Emotion recognition	Emotiv; OpenBCI; NeuroSky MindWave
Asl et al.	2022	Drowsiness detection	MyndPlay Mindband; NeuroSky MindWave; Emotiv

*Note*. These reviews were excluded from the present review for reasons outlined in Method and Results.

While these non-systematic reviews provide insights into the domain-*specific* functions of certain EEG devices, the current literature on this topic is, at best, fragmented. Indeed, it is surprising that, to date, there has been no systematic scoping review on the research-related use of currently available and commonly used consumer-grade EEG devices. Thus, the aim of this paper is to chart the large volume of studies that have used consumer-grade EEG to collect neural data. We categorise each of these studies by domain or category to provide a domain-*general* snapshot of the work conducted with this emerging technology. Note that the purpose of this scoping review is to detail the range of consumer-grade EEG usage in the current literature. We do not provide an exhaustive critical analysis of each device, as per the remit of a scoping review in [32].

## Method and results

We followed the Preferred Reporting Items for Systematic Reviews and Meta-analyses Extension for Scoping Reviews (PRISMA-ScR) guidelines [32]. Scoping reviews report the “extent, range, and nature of research activity” [p. 21] in five stages: (1) identify the research question; (2) identify relevant studies; (3) select the studies; (4) chart the data; (5) collate, summarise, and report the results.

### Step 1: Identify the research question

We aimed to explore the extent to which consumer-grade EEG has been applied to research domains in locations around the world. Regarding EEG devices, our goal was to identify the EEG systems produced by companies that manufacture the most commonly used consumer-grade devices. Regarding research domains, our goal was to identify the primary research fields that these consumer-grade EEG systems have been applied to. Finally, regarding research location, our goal was to answer the question of where the devices have been used globally.

### Stage 2: Identifying relevant studies

We conducted a systematic literature search by retrieving records from five online databases: (a) PsycINFO, (b) MEDLINE, (c) Embase, (d) Web of Science, and (e) IEEE Xplore. These databases contain studies from multiple fields including neuroscience, psychology, medicine, and engineering. Searches included studies published from 2010, as this is the year the first studies examining consumer-grade EEG were published. Studies also had to be conducted with human subjects and written in English. To find records in each database, we used search strings to capture keyword variations (see Table 2). These were subsequently adjusted to fit the search syntax of each database.

**Table 2:** Search strings used for each device.

Device	Device-specific Search String	Domain-general Search String
Emotiv	(“emotiv” or “epoc”)	(“electroenceph*” or “electrophys*” or “eeg” or “event-related” or “event related” or “erp”)
interaXon Muse	(“interaxon” or “muse”)	
Myndplay Mindband	(“myndplay” or “mindband”)	
NeuroSky MindWave	(“neurosky” or “mindwave”)	
OpenBCI	(“openbci” or “cyton” or “daisy”)	

*Note*. Each device-specific search string was linked to the domain-general search string using the “AND” logical operator. Asterisks indicate truncation wildcards.

### Stage 3: Study selection

**Fig 1:**
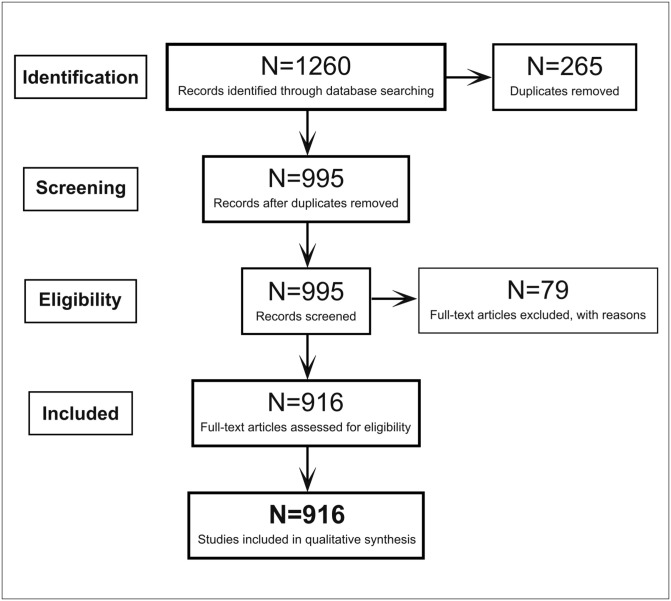
PRISMA flowchart of the screening process.

The initial search was conducted in April 2022, completed in May 2022, and yielded 1260 articles. We excluded 265 duplicates and screened the remaining using systematic review software, SYRAS [34]. After screening, we excluded a further 79 studies as they met one or more of the following exclusion criteria: no device was used, or no EEG was data collected (*N* = 53); not written in English (*N* = 6); had inaccessible PDFs (*N* = 20). The final number included was 916, consisting of 531 journal articles and 385 conference proceedings. Fig 1 depicts the flow chart of the screening process.

### Stage 4: Charting the data

We charted 916 included articles by recording the following information: EEG device, research domain, authors, first author’s country, year of publication. Country information was obtained from the first author’s affiliation as listed in the article. If this could not be found, the first author’s most recent affilation was used.

### Stage 5: Collating, summarising, and reporting the results

#### Consumer-grade EEG devices

The devices used by articles included in this scoping review were produced by five companies: **Emotiv** (67.69%), **NeuroSky MindWave** (24.56%), and **OpenBCI**, **interaXon**, and **MyndPlay** (7.75% collectively for these three devices). **Emotiv** has released three versions of a 14-channel EEG device: EPOC, EPOC+, and EPOC-X. For simplicity, all three devices will hereon be referred to as “Emotiv.” The EPOC+ and EPOC-X can capture data at both 128 and 256 Hz sampling rates, whereas EPOC captures data at 128 Hz. The EPOC-X includes an updated amplifier configuration, sensor housing, and rotating headband. **OpenBCI** provides two highly customisable EEG systems: the four-electrode Ganglion board and the eight-electrode Cyton board. Again, for simplicity, both boards will hereon be referred to as “OpenBCI.” Users can configure an assembly of eight electrodes around an OpenBCI Cyton processing circuit board and daisy-chain it to another Cyton board for a 16-channel system. The **interaXon Muse** is a headband-shaped device that records at 256 Hz from four electrodes located above the eyes (AF7, AF8) and above the ears (TP9, TP10). The **MyndPlay MyndBand** records at 512 Hz from three electrodes on the forehead. The **NeuroSky MindWave** is shaped like a gaming headset, records at 512 Hz from a single electrode on the forehead (FP1), and pairs with a mobile application that can detect power spectra and other metrics such as meditation and attention using built-in signal processing techniques.

Research and consumer-grade EEG devices typically differ in their electrode count. Whereas research-grade EEG devices typically comprise dozens of electrodes fitted over the scalp with saline gel or solution, consumer-grade EEG devices have fewer electrodes and use minimal or no conductive medium. However, despite their low electrode count, consumer-grade EEG devices have been validated for scientific research in a broad range of paradigms. Table 3 lists the technical specifications of currently available devices.

**Table 3:** Technical specifications of all currently available commercial EEG devices.

Manufacturer	Model	Release Year	Channels	Sample rate (Hz)	Resolution
Emotiv	EPOC+	2013	14	128 or 256	14-bit
MyndPlay	Mindband	2014	1	512	*
OpenBCI	Ganglion board	2014	4	200	24-bit
OpenBCI	Cyton board	2014	8	250	24-bit
OpenBCI	Cyton-Daisy board	2014	16	125	24-bit
Emotiv	Insight	2015	5	128	14-bit
InteraXon	Muse 2	2016	4	256	12-bit
NeuroSky	MindWave Mobile 2	2018	1	512	12-bit
Emotiv	EPOC-X	2019	14	128 or 256	14-bit

*Note*. Emotiv also manufactures the EPOC Flex that contains 32 channels. This device is not included as it is explicitly marketed as a *research* device—not a commercial or consumer-grade device.

#### Research domains

**Fig 2:**
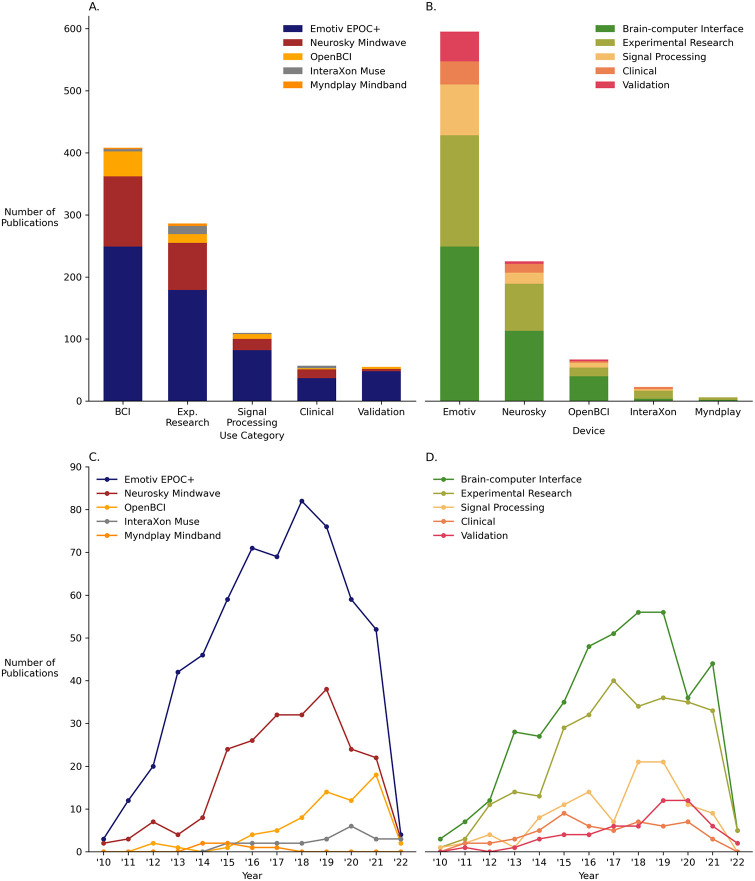
Charting the number of publications for each device and usage category. *Note*. For all plots, the *y*-axis denotes the number of publications per category. (A) depicts a bar plot of the total number of publications categorised by usage and subcategorised by device in a single bar. (B) charts the inverse: total number of publications categorised by device and subcategorised by usage in a single bar. (C) and (D) are frequency plots that chart the number of publications over time since 2010 by device and usage, respectively.

The included articles fell into five categories of use: *BCI*, *experimental research*, *validation*, *signal processing*, and *clinical*. Table 4 lists a description and an example for each usage category, and Fig 2 provides a visual depiction of device usage by category and over time. It should be noted that these categories are not mutually exclusive. For example, a study that compared the performance of inexpensive, wireless, and dry EEG systems in classifying common neural responses [35] would be “validation.” In cases where a device could be classified under multiple categories, the authors made a joint decision about the most appropriate category.

**Table 4:** Usage category descriptions and examples.

Usage Category	Description	Example
Brain-computer Interface (BCI)	Studies that facilitated interaction between human user and computer (or machine).	Realtime control of a wheelchair.
Experimental Research	Studies that used the device as a theory development tool	Examining EEG signatures in cognitive tasks.
Validation	Studies that aimed to validate use of the device	Comparing consumer-grade to research-grade EEG systems.
Signal Processing	Studies that collected data to refine EEG signals.	Creating a classification algorithm to reduce artefacts.
Clinical	Studies that used the device as a clinical tool	Assessing drowsiness in drivers.

*Brain-computer interfaces*. The majority (44.54%) of included studies used consumer-grade EEG as an interface between a human user and computer. Through the application of machine learning algorithms, users are given the ability to control computers (software) or computer-based machines (hardware) and perform a variety of functions [27]. This is typically achieved in two steps. Firstly, data from a user is collected to train a classifier to recognise and extract features (i.e., spikes or longer pattern irregularities) from a continuous EEG signal, usually in response to a target visual or auditory stimulus. These features can then be evoked by the user to control a configured BCI system. How well the BCI system performs depends on multiple factors that include the chosen algorithm, the quality of the signal, electrode location, and the feature to be extracted [36–38].

The two earliest BCI-based studies were published in 2010. Using Emotiv, Ranky and Adamovich [39] trained subjects over three weeks to move a robotic arm in three dimensions and grip certain objects using only facial movements. Likewise, Kwang-Ok et al. [40] used Emotiv to collect data for a paraplegic subject’s use of an emergency call system. These early studies speak to the functional and clinical applicability of portable BCI systems, particularly for communication with and the motor improvement of paralysed patients.

One of the most common methods to configure a BCI system using consumer-grade EEG is with the steady-state visual evoked potential (SSVEP). SSVEPs are the typical neural response to visual stimuli that appear at matching frequencies [41]. In a recent study, Garcia et al. [42] used the interaXon Muse and SSVEPs (along with other signal processing algorithms) to decode and reconstruct stimuli in subjects’ visual field with remarkable accuracy. In a similar study, Shi et al. [43] examined SSVEP image decoding applied to mobile phones. Chumerin et al. [44] designed a maze-navigating game that used SSVEPs and Emotiv; and, together with an eye-tracker, Brennan et al. [45] used Emotiv to control a smart home with SSVEPs. Given the frequent use of SSVEPs, future researchers may also choose to take advantage of this feature’s high signal-to-noise ratio when implementing consumer-grade EEG into BCI systems.

Another easily identifiable ERP feature is the P300, a positive spike occurring roughly 300 ms after the presentation of an irregular, or “oddball”, stimulus in a stream of otherwise regular stimuli [46]. Due to its consistent latency, the P300 is commonly used as a target response in BCI-based applications [47]. Jijun et al. [48] used an Emotiv to capture P300 components and created a hands-free dialling system. Indeed, one of the most frequently observed applications of consumer-grade EEG is in P300 speller paradigms [49–53]. To illustrate, a matrix of letters is shown to an observer. To spell out a word, the observer must sequentially focus their attention on a single letter while each row and column in the matrix is rapidly highlighted. As one can imagine, combined with a portable device, P300 spellers prove extremely useful for those with verbal and motor difficulties.

Motor-imagery is a purely mental process that involves the imagined execution of motion without explicit muscular or peripheral action [54]. There has been almost three decades of research involving BCI systems and motor imagery [55, 56], with more recent implementations using consumer-grade EEG. By training an algorithm to detect specific EEG patterns reflecting imagined motor functions, users can operate artificial limbs [39, 57–61] and wheelchairs [62–67]. Motor imagery with consumer-grade EEG has also been used to control electric vehicles [68] and aerial drones [8, 69, 70]. Parikh and George [71] designed a BCI system that uses motor imagery to control a quadcopter, and Das et al. [72] designed a BCI system to control a quadcopter with a user’s directional intentions.

Some of the most innovative BCI applications involved an interaction between a human user and software, rather than hardware. Shankar and Rai [73] used Emotiv to facilitate 3D computer-aided design (CAD) modelling, where user-evoked responses can activate various commands. Consumer-grade EEG has also been used to control web browsers [12]. Yehia et al. [74], for example, designed an SSVEP-based website interface that presents options that can be selected with visual attention according to available functions on the current page.

More recent studies have explored the use of consumer-grade EEG in biometrics. Much like a fingerprint or password, algorithms can identify individuals based on neural patterns: Moctezuma et al. [75] identified subjects via their EEG responses using imagined speech; Do et al. [76] identified subjects according to their responses to images; and Saini et al. [77] collected responses while subjects handwrote signatures to generate unique individual biomarkers.

*Experimental research*. Studies classified under experimental research used consumer-grade devices to collect EEG data outside of BCI applications. These studies comprised 31.22% of included articles and spanned a wide range of paradigms. For instance, the Emotiv was used in studies examining pilots’ reactions to unexpected events [78], reaction time [79], and mental fatigue and alertness [80]. Similarly, many experimental studies aimed to detect drowsy states [16, 81–85]. Such paradigms have been adopted to improve road safety with the creation of early warning systems that alert drivers to their own fatigue before an accident can occur [86–89]. These studies expand the application of EEG outside the laboratory for purely experimental purposes. Further studies have been conducted with consumer-grade EEG on students in the classroom [90–92]. Dikker et al. [93] used the Emotiv to measure the synchronised neural activity across students in a class, and found that this measure predicted overall classroom engagement and social dynamics.

Consumer-grade EEG has also been used to augment experiments testing athletes’ performance and mental states. Borisov et al. [94] used the Emotiv together with other biological indicators to examine athletes’ attention and stress levels. Liu et al. [95] correlated rifle-shooting accuracy with EEG signatures to find optimal states for good shots. Using the NeuroSky MindWave, Azunny et al. [96] found that meditation moderated athletes’ attention and working memory. Finally, Sultanov and Ismailova [97] determined power spectra during football players’ training and resting states.

While these studies report the performance of consumer-grade EEG across different experimental paradigms, we advise caution in applying consumer-grade devices to experiments that require excessive motion. EEG signals are inherently noisy. In cognitive and perceptual studies, researchers must typically remove trials where a subject’s movements introduce artefacts. In extreme cases, to avoid spurious results, a large portion of subjects must be removed from analyses due to such artefacts. There are many in-house tutorials for popular toolboxes such as EEGLAB [98] and MNE [99], amongst other best-practice guidelines for cleaning EEG data [100–103]. We refer the reader to these guidelines to ensure the collection of high-quality neural signals for publication and replication.

*Signal processing*. Signal processing studies comprised 12.01% of included articles. These were studies that used consumer-grade EEG to create or improve signal processing algorithms or pipelines. Multiple studies have used an array of clustering and component analytic methods to remove eyeblinks and clean the signal recorded with consumer-grade EEG [104–111]. For instance, Szibbo et al. [112] proposed a novel algorithm to remove blink artefacts with logarithmic smoothing methods. Trigui et al. [113] used the Emotiv to accurately detect SSVEPs with the multivariate synchronisation index, an algorithm that characterises the synchronisation between recording and reference electrodes. Trigui et al. [114] also used the Emotiv to evaluate classification accuracy using inter-battery factor analysis. While the authors did not test for significance, this method produced numerically greater accuracy compared to two other methods: canonical correlation analysis and multivariate synchronisation index. Finally, Elsawy et al. [115] proposed a principal components analysis classifier to use with P300 spellers.

Given the relatively lower signal quality of consumer-grade compared to research-grade EEG devices, future research aiming to improve signal processing methods may apply existing algorithms to data collected with research-grade EEG devices. This can assist further experimental EEG work by validating the algorithms’ robustness when applied to higher-quality data.

*Clinical*. Clinical experiments comprised 6.22% of included articles. These studies involved the diagnosis and treatment of various physical and mental illnesses. Three recent clinical studies show reliable applications in stroke patients. After observing good test-retest reliability [116], Rogers et al. used the NeuroSky MindWave to determine distinct EEG profiles for patients who have experienced transient ischemic attack, ischemic stroke, or neither [117]; Wilkinson et al. [118] used the interaXon Muse to accurately identify patients with large vessel occlusions, a diagnostic predictor of acute stroke; and Ishaque et al. [119] used the interaXon Muse to characterise neuronal function and clinical recovery of stroke patients post-treatment.

Recording abnormal neural responses to stimuli can further assist in creating clinical profiles. Terracciano et al. [120] designed smart glasses configured with an OpenBCI Cyton board to generate a distinct visual checkerboard pattern that elicits the pattern-reversal visual-evoked potential in neurotypical populations. The onset of this component is significantly delayed in multiple sclerosis and traumatic brain injury [121]. Consumer-grade devices coupled with diagnostic tools can facilitate more accurate diagnoses, evident from studies on insomnia [122], attention-deficit hyperactivity disorder [123], and epilepsy [124].

Seizure detection is a particularly promising application for consumer-grade EEG [125–127]. Signals collected by EEG devices can employ a variety of signal processing techniques and provide convergent evidence of early seizure onset. While this application is in its early stages (see [128]), seizure detection is possible by using algorithms that are trained on an epileptic patient’s profile. In future, perhaps a portable alert system combined with an EEG headband or headset can warn patients (and others nearby) to move to a safe place prior to seizure onset.

Lastly, a potential clinical application for consumer-grade EEG devices is in neurofeedback therapy. Neurofeedback is a form of operant conditioning that involves showing a patient their neural signals in real time to reinforce optimal and extinguish negative mental states [129]. Using consumer-grade EEG devices, various neurofeedback systems have been designed for the treatment of Parkinson’s disease [13], central neuropathic pain [130], attention-deficit hyperactivity disorder [131, 132], dyslexia [133], and depression [134].

*Validation*. Validation studies comprised 6.18% of included articles in this review. The most informative of these involved comparing recordings between consumer and research-grade EEG devices. Grummett et al. [35] report that the Emotiv could reliably demonstrate the Berger effect (relative increase in alpha wave amplitude from electrodes placed above occipital and parietal cortex) compared to other inexpensive, wireless, and dry EEG systems. They also found that the Emotiv’s capacity to detect visual steady-state responses was comparable to a research-grade g.Hlamp. However, the Emotiv displayed a significantly higher noise floor than other systems, prompting the authors to caution its use in studies that examine lower frequency spectra. Kotowski et al. [135] reported reliable measurement of the early-posterior negativity when subjects responded to different emotional stimuli. Multiple studies comparing the Emotiv to a Neuroscan system provide evidence that the Emotiv performs remarkably well at recording research-grade ERPs in multi-trial experiments. De Lissa et al. [136] observed reliable recordings of the face-sensitive N170 component. Badcock et al. [137] found that the Emotiv can reliably record a series of late auditory ERP components: P100, N100, P200, N200 and P300. Barham et al. [138] found that N200 and P300 did not differ between the two systems—though, they also found that significantly more trials were rejected from the Emotiv during preprocessing. Additionally, Ries et al. [139] found that the Emotiv was comparable to a research-grade BioSemi ActiveTwo after correcting for low frequencies. In terms of event timing, Hairston et al. [140] observed only slight jitter and delay in the Emotiv’s serially-ported event timing, while Williams et al. [141] recorded comparable event timing between Emotiv and Neuroscan.

Only a single study reported disparities between the waveforms of the Emotiv and a medical-grade Advanced Neuro Technology system: Duvinage et al. [142] found differences in their recording of P300 responses. However, as might be expected, consumer-grade EEG devices display considerably poorer signal-to-noise ratios than their research-grade counterparts. Mahdid et al. [143] found that the functional connectivity of both Emotiv and OpenBCI systems compared poorly to research-grade EEG systems; Raduntz [144] found that the signal reliability of the Emotiv was poor if the device did not exactly fit the subject’s head; and Ekandem et al. [145] observed that the signal quality of an Emotiv, specifically the EPOC+, declines over time as it uses a built-in rechargeable battery.

There were few validation studies for the remaining devices. Two studies compared the NeuroSky MindWave’s performance to research-grade systems. Johnstone et al. [146] found very minor differences in signal quality between a NeuroSky MindWave and a Neuroscan; and Rogers et al. [116] found good test-retest reliability in eyes-closed paradigms, with slightly lower reliability in eyes-open paradigms. Frey [147] compared the OpenBCI’s spectral and temporal features to a medical-grade g.USBamp and found negligible differences. No validation studies were found for the MyndPlay Mindband or the interaXon Muse.

#### Research location

**Fig 3:**
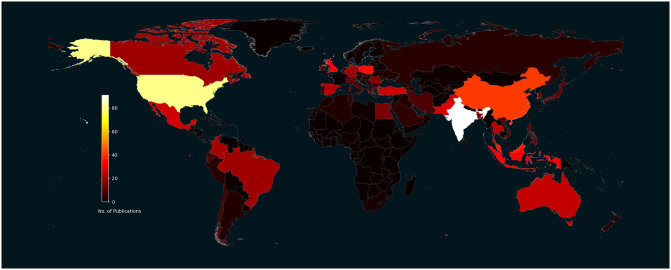
Geographical heatmap of publications that used consumer-grade EEG for research by country. *Note*. This heatmap shows the number of consumer-grade EEG articles published in each country. We recommend viewing this paper online for the full colour version the Fig 3.

Fig 3 shows a geographical heatmap of the number of publications by each country. In sum, the 916 studies that used consumer-grade EEG since 2010 were conducted across 83 countries spread across six continents. The top ten countries accounted for 46.72% of included studies. In order, these were: India (9.72%), United States (8.73%), China (4.48%), Malaysia (4.04%), Poland (3.82%), Indonesia (3.49%), United Kingdom (3.49%), Pakistan (3.06%), Mexico (2.95%), and Turkey (2.95%).

## Discussion

The aim of this scoping review was to explore the extent to which consumer-grade EEG devices have been applied to research domains in locations around the world. We identified 916 peer-reviewed studies that used consumer-grade EEG to collect neural data from human subjects. We expand on device-specific information, location, and usage across research domains in turn.

Regarding **consumer-grade EEG devices**, Emotiv devices were the most widely used. We speculate that this was primarily due to its easy setup, relative comfort compared to other devices, and capacity to record quality ERPs. Emotiv devices require no engineering or technical expertise and configuring event markers is relatively easy using a serial port cable. However, there are a few caveats researchers should be aware of when using Emotiv. Firstly, the device has quite a low noise floor. We do not recommend using Emotiv for single-trial experiments. We also advise against using Emotiv in studies that require localising activity (e.g., functional connectivity experiments), as the plastic arms do not accommodate every head shape or size. Beyond these caveats, the Emotiv has proven useful in experiments that involve multiple trials: psychophysical or cognitive tasks. Additionally, by applying task-appropriate algorithms, users can compensate for the Emotiv’s low noise floor, and the device can be implemented in BCI systems.

Despite only having a single electrode, the NeuroSky MindWave has also been widely used for research. The NeuroSky MindWave was particularly popular in BCI systems, market research, and gamification. However, there is conflicting evidence for its utility. Maskeliunas et al. [148] found that the MindWave displays poor accuracy at recognising the meditative and attentive states it claims to have built-in metrics for. Meanwhile, Rogers et al. [149] found that the device can assist in predicting functional outcomes after stroke. Thus, the NeuroSky MindWave may be useful in experiments that strictly examine the time-course of recordings from FP1 as its signal quality has been shown to be equal to or even greater than an Emotiv device. Further, the MindWave is an affordable EEG device for clinicians to complement data from other diagnostic metrics. These are rich avenues for future use of the MindWave as a research tool.

As for the three remaining devices, it is evident that more research is required to explore and perhaps validate their further experimental use. While OpenBCI has contributed to studies in engineering and robotics, more experiments are needed for an informed evaluation of OpenBCI’s applicability to cognitive neuroscience. Both the interaXon Muse and the MyndPlay Mindband have been used in general experimental and clinical studies. Future research may address this notable gap in the literature by comparing these under-utilised devices to research-grade EEG devices. Validation studies, in particular, can present the advantages and challenges associated with each device and pave the way for better-informed, specialised application of consumer-grade EEG devices. From our review, addressing the most notable research gap would involve more well-powered within-subjects studies that compare signal-noise indices for each device. Future researchers may find Table 5 useful, as it highlights some general recommendations from our synthesis.

**Table 5:** General recommendations for each device.

Device	Key Advantages	Disadvantages
Emotiv	Useful in cognitive tasks involving multiple trials; records quality ERPs.	Poor localisation; low noise floor; requires rigorous preprocessing pipeline to yield clean signal.
interaXon Muse	Potential use in clinical/therapeutic applications.	Requires further validation research.
Myndplay Mindband	Comfortable–designed like a gaming headset.	Requires further validation research.
NeuroSky MindWave	Can record relatively accurate signals from FP1; comfortable.	No reference electrodes for component analytic preprocessing.
OpenBCI	Up to 16 electrodes and flexible configuration; documented effectiveness in BCI control.	Requires further validation research.

*Note*. These recommendations are overarching themes that became apparent through the present charting. A more rigorous investigation regarding signal comparison and application to different paradigms is required for a detailed understanding of each individual device’s capabilities.

Focusing now on **research domains**, this review revealed that the primary research-related use for consumer-grade EEG devices is for BCI systems. This may be due to the relatively low cost of administering large-*N* studies with these devices. Additionally, given enough training data, probabilistic models can now be configured for an individual output signal with impressive accuracy [150, 151]. With more powerful computers and better classification algorithms, we expect that this application will only continue to grow as neuroscientists increasingly use machine learning to solve problems.

The second most frequent research-related use was for general experimental research, spanning from cognitive tasks and recording athletic performance to low-level auditory or visual perception studies. This is further evidence that the scope of consumer-grade EEG includes a wide range of fields. Perhaps future research may focus on adding to the relatively low number of validation and signal processing studies, as these are crucial in informing users of the devices’ performance across different contexts. We also highlight the potential for consumer-grade EEG devices to be applied to clinical studies. We observe that BCI research has provided evidence of the devices’ proficiency in creating biometric profiles for users, as well as prototypes for artificial robotic limbs, drowsiness detection systems, and mobility aides. With the advent of further clinical research, we may see more frequent use of the devices in hospitals and clinics, where neural augmentation has the potential to improve patient identification, diagnosis, and treatment outcomes.

As well as providing information about the scope of consumer-grade EEG devices, research domains, and research location, we note that the studies included in this review revealed an interesting trend in terms of date of publication: The number of publications using consumer-grade EEG devices peaked in 2018, then dropped slightly in 2019 the year before the COVID-19 pandemic. During the peak years of the pandemic in 2020 and 2021, this number dropped substantially (see Fig 3C and 3D). This is unsurprising, as researchers’ ability to administer EEG studies to human subjects was adversely affected by the imposition of lockdowns and travel restrictions worldwide [152–154]. While the number of studies using consumer-grade EEG devices was reduced during the pandemic, few EEG studies continued globally in diagnostic COVID-19 experiments [155–157]. If consumer-grade EEG devices were more well-recognised by the wider scientific community, the devices may have found utility in large-scale EEG experiments during a time when such neural data could augment epidemiological studies [158]. They may also have proved useful to cutting costs associated with the usage and maintenance of research-grade machines. This is particularly true for Emotiv systems as the company provides a platform for the construction, deployment, and data collection of online EEG experiments [159].

Regarding **research location**, it was encouraging to discover that the use of consumer-grade EEG devices is not limited to Western, industrialised countries [160, 161]. We found that studies were reasonably spread across multiple countries. The same consumer-grade EEG device used across different locations can record with roughly equivalent quality, which increases the reliability of replication EEG studies.

A core limitation of our review is the use of five databases: unlisted articles would have been missed. As such, we may not have included the full scope of studies that used consumer-grade EEG. We also only included articles published in English, and articles referring to their respective consumer-grade EEG device by a different name would also have been missed. Finally, we acknowledge the limitations of a scoping review in general in not aiming to provide detailed criticism nor inferences on charted articles. Given the high number of consumer-grade EEG articles, this paper instead provides grounds for a future meta-analysis to subset from this wide range, collect effect-sizes, and compare device signal quality within specific experimental paradigms.

## Conclusion

Despite being marketed for commercial purposes, we present evidence that consumer-grade EEG devices have found considerable utility in scientific research. These devices have been used in non-traditional research settings and applied in innovative ways. In this paper, we provide a structured review of the current state of the literature and provide some general guidelines for their scope of use, aggregated from a large subset of consumer-grade EEG studies. Significant and impactful future research for consumer-grade EEG span multiple fields from cognitive neuroscience to engineering. Consumer-grade EEG can be used for BCI research, as well as multi-trial experimental, clinical, and validation paradigms that take advantage of the devices’ relative portability and affordability.

From these observations, affordable and accessible neuroscientific solutions are becoming more available to those outside of research spheres. The use of consumer-grade EEG devices is a particularly salient issue given our increasingly technologically augmented lifestyles. Indeed, it is not outside the realm of possibility to see EEG being used in everyday life within the next few decades. Thus, to help inform scientists, practitioners, and the general public on the appropriate use of consumer-grade EEG devices, we encourage researchers to further explore the capabilities of this technology.
